# Diagnosis of Streptococcus suis Meningoencephalitis with metagenomic next-generation sequencing of the cerebrospinal fluid: a case report with literature review

**DOI:** 10.1186/s12879-020-05621-3

**Published:** 2020-11-25

**Authors:** Xiaobo Zhang, Zhaoping Wu, Kai Wang

**Affiliations:** 1 Department of Neurology, The First People’s Hospital Of Changde City, 818 Renmin Road, Changde, 415000 Hunan Province China; 2 Department of Neurology, Second Xiangya Hospital, Central South University, Changsha, 410011 Hunan Province China

**Keywords:** Streptococcus suis, Meningoencephalitis, Cerebrospinal fluid, Metagenomic next-generation sequencing

## Abstract

**Background:**

Streptococcus suis meningoencephalitis is a zoonotic disease that mostly infects slaughterhouse workers. Rapid diagnosis of Streptococcus suis meningoencephalitis is critical for effective clinical management of this condition. However, the current diagnostic techniques are not effective for early diagnosis of this condition. To the best of our knowledge, the use of cerebrospinal fluid metagenomic next generation sequencing in the diagnosis of Streptococcus suis meningoencephalitis has been rarely reported.

**Case presentation:**

Here, we report a case of Streptococcus suis meningoencephalitis in a 51-year-old female patient. The patient had a history of long-term contact with pork and had a three-centimeter-long wound on her left leg prior to disease onset. Conventional tests, including blood culture, gram staining and cerebrospinal fluid culture, did not reveal bacterial infection. However, Streptococcus suis was detected in cerebrospinal fluid using metagenomic next generation sequencing.

**Conclusions:**

Metagenomic next generation sequencing is a promising approach for early diagnosis of central nervous system infections. This case report indicates that cases of clinical meningeal encephalitis of unknown cause can be diagnosed through this method.

## Background

Streptococcus suis (S. suis) is a zoonotic pathogen that may cause serious illness in pigs and people who have occupational contact with pigs and pork, such as farmers, slaughterhouse workers and butchers [1]. In humans, S. suis infection may cause serious infections and complications including meningitis and septicemia. Meningitis is the most frequent clinical manifestation of *S. suis* infection, and deafness is the most common sequelae in survivors [2, 3]. About 50–60% of S. suis infections occur even in the absence of obvious wounds after contact with pigs or pork, and may be asymptomatic in the early stages. Moreover, disease progression is rapid, and the pathogen may directly enter circulation causing meningeal encephalitis [2, 4]. However, the clinical incidence of *S.* suis meningitis is very low (3%) which often leads to misdiagnosis of this condition, especially in the context of pneumococcal meningitis (20%) and *Listeria monocytogenes* meningitis (36%) [5, 6]. Thus, there is a need to develop accurate methods for rapid detection of S. suis to promote effective treatment. Routine microbiological tests are often insensitive against neuroinvasive pathogens. Metagenomic next generation sequencing (mNGS) is a promising, universal pathogen detection method which provides “unbiased” pathogen detection. It can, therefore, be used for the clinical diagnosis of various pathogens, including rare ones [7, 8]. It is a more accurate method since it relies on DNA sequence information contained in cerebrospinal fluid samples of patients with meningitis or encephalitis [7, 9].

Here, we report a case of *S. suis* meningoencephalitis which was diagnosed with mNGS analysis of cerebrospinal fluid samples. To the best of our knowledge, there are few reports on the application of mNGS to detect pathogens in cerebrospinal fluid (CSF) of patients with S. suis meningoencephalitis**.** The study was approved by the ethics committee of the First People’s Hospital of Changde city and publication of the clinical data was approved by the patient’s family.

## Case presentation

Here, we report a case of a 51-year-old female diagnosed with *S. suis* meningoencephalitis.

The patient was a slaughterhouse worker and had no chronic illnesses. In addition, the patient had no history of alcohol or illicit drug use, but had a three-centimeter-long wound on her left leg before disease onset. The patient presented to our emergency department with a 3-day fever, with a temperature of nearly 39.0 °C.

At the time, the patient did not experience any other symptoms, such as cough, expectoration, or chills. Initially, the patient was treated for putative infection at a local clinic (specific treatment information was not available), but her symptoms did not improve significantly. On the 3rd day of symptom onset, she experienced an occipital headache of unknown nature and duration, accompanied by dizziness, nausea and vomiting, without obvious limb weakness, incoherent speech, abnormal mental behavior, convulsion or other discomfort. The patient was transferred to our hospital’s emergency department for further treatment. Routine blood analysis at the emergency department revealed a WBC count of 10.56 × 10^9/L, N%: 94.4%. A head CT scan did not reveal any obvious abnormality. Within 24 h, the patient was admitted into the intensive care unit. In the morning after admission, a lumbar puncture was performed which showed turbid CSF. The CSF pressure was 310 (mmH2O) and the color of the CSF was light yellow, with slight turbidity. The WBC count was 850/μLand the WBCs were multinucleated. Biochemical analysis of the CSF revealed that the levels of glucose, chloride and protein were 0.15 mmol/L, 117.5 mmol/L, and 4275 mg/L, respectively. About 5 mL of the CSF was sent to KingMed Diagnostics for CSF pathogen identification using next generation sequencing. At this time, the patient was treated with meropenem. A repeat lumbar puncture 5 days after symptom onset revealed relatively clear CSF and lower CSF pressure relative to the previous value. CSF WBC count also dropped to 680/μL. Biochemical studies demonstrated that the levels of glucose, chloride, and protein were 4.18 mmol/L, 124.9 mmol/L, and 1324 mg/L, respectively. Cranial MRI revealed a wide range of enhancement patterns. Viral and tuberculosis tests were negative.

**Fig. 1:**
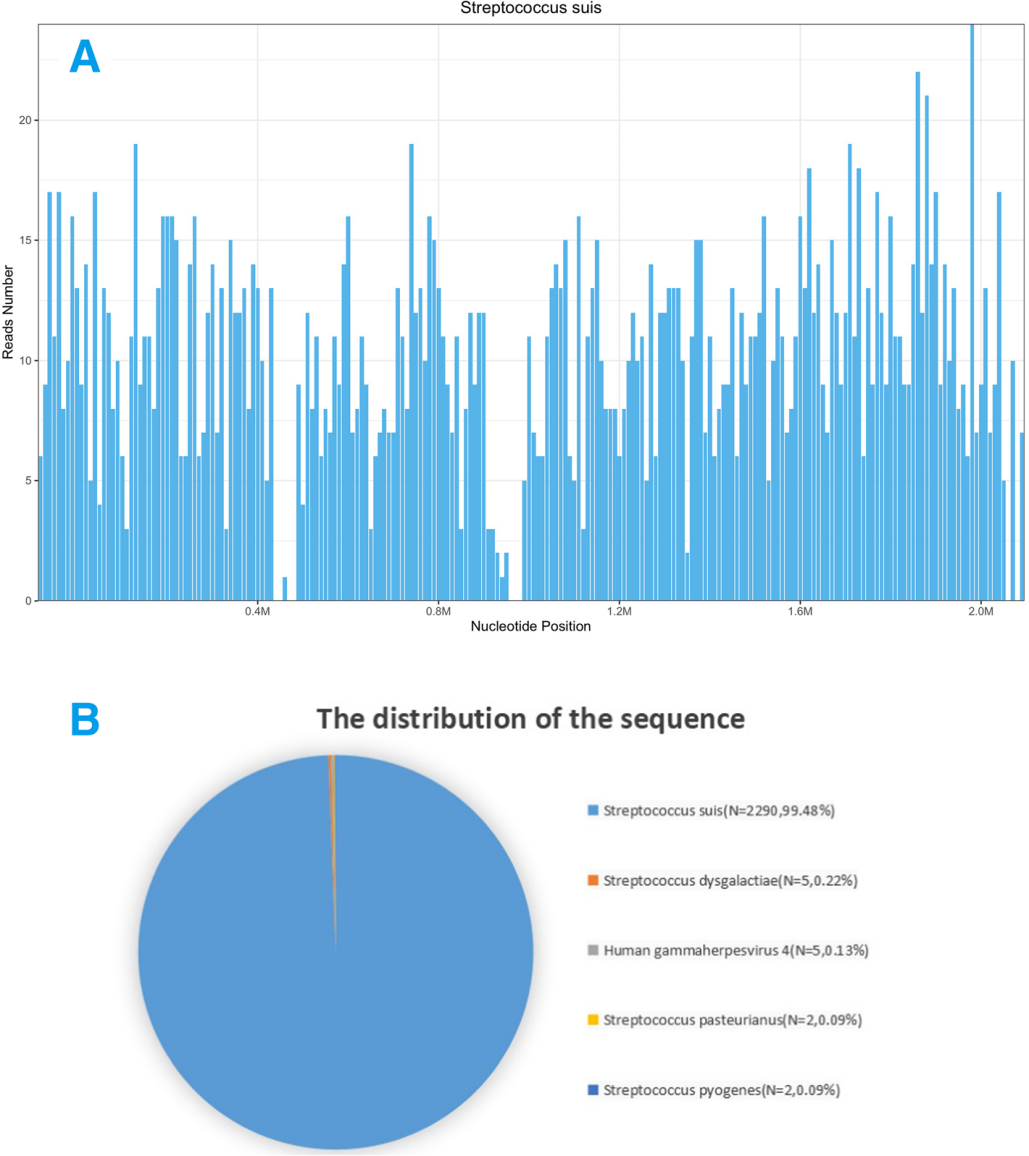
The majority of reads mapped to the S. suis genome, with coverage of 5.5508% (**a**). A total of 2290 sequence reads corresponded to S. suis, accounting for 99.48% of microbial reads (**b**)

Four days after admission, analysis for wide-spectrum bacterial nucleic acids did not detect bacteria in the CSF. By contrast, CSF mNGS analysis confirmed the presence of *S. suis* with a very large number of sequences (Fig. 1). Very low levels of *Streptococcus dysgalactiae* were also detected, which was more likely to be non-specific matching of homologous sequences. Studies have shown that *Streptococcus dysgalactiae* can colonize the skin of humans hence may cause CSF contamination [10]. Together with medical history, we concluded that the patient was infected by S. suis. The patient’s EEG showed diffuse slow waves, with no epileptic wave. Repeated lumbar puncture 9 days after admission revealed a CSF pressure of <180mmH2O, WBC count of 40/ul, a glucose level of 3.23 mmol/L, a chloride determination level of 123.2 mmol/L, and a protein level of 703.0 mg/L, indicating marked patient improvement. The results of 4 CSF tests are shown in Table 1.

**Table 1:** The results of 4 times cerebrospinal fluid of the patient after admission in our hospital

Date	Pressure (mmH2O)	White blood cell (count/ul)	Protein (mg/L)	Glucose (mmol/L)	Chloride (mmol/L)
Day 3 of symptom onset	310	850	4275	0.15	117.5
Day 5 of symptom onset	280	650	1324	2.68	124.9
Day 12 of symptom onset	70	40	703	3.23	123.4
Day 20 of symptom onset	90	3	325	3.68	125.7

On the 10th day after admission, the patient was transferred to the general ward and treated with ceftazidime in the subsequent days. Since then, she complained of dizziness and hearing loss. We have attempted to try to correct the hearing loss of the patient by some drugs such as hormones and vitamins, but the effect was not obvious. Repeat lumbar puncture 20 days after symptom onset revealed completely normal values for routine and biochemical CSF analyses. The patient’s physical condition also gradually improved and asked to be discharged from hospital. The patient still had some hearing loss at the time of discharge.

During hospitalization, bacterial culture and gram staining of CSF showed normal results. Repeated CSF acid-fast staining was also negative. This indicates that mNGS is more effective than conventional microbial detection methods. Due to its high costs, mNGS reanalysis was not done. At one-month follow-up, the patient still complained of left hearing loss but could function independently.

## Discussion and conclusions

Although intracranial infections are common, the pathogens are not identified in 50% of intracranial infection cases [11]. The bacteria most commonly associated with central nervous system infection and serious intracranial infection include, Neisseria meningitidis and *Streptococcus pneumoniae* [12]. Streptococcus suis (S. suis) has been reported as a novel emerging zoonotic pathogen. The main risk factors for S. suis infection include occupations related to pigs, exposure to pigs or pork products, and being male [2, 3]. This pathogen is naturally present in piglets and can cause serious human illnesses, including meningitis, endocarditis and septicemia [2, 4]. It has been reported that most cases of *S. suis* meningitis are male. Typical clinical manifestations include hearing loss, fever, headache, and stiff neck, but hearing loss is the most frequent [2, 13].

The female patient in this case report presented with fever and headache as first symptoms, which were accompanied with dizziness and unconsciousness. The patient complained of hearing loss during hospitalization. Repeat CSF smear tests, common cultures and CSF broad-spectrum bacterial nucleic acid tests were negative during hospitalization. For quick, accurate diagnosis, mNGS analysis of CSF was performed which indentified S. suis.

To detect pathogenic microorganisms by mNGS, parallel sequencing of all nucleic acids (DNA and/or RNA) from samples is performed [14, 15]. Although standard microbiological diagnostic techniques fail to detect pathogens in most cases [16–18], unbiased mNGS application successfully identifies causative pathogens in such cases, making mNGS a superior diagnostic tool [14, 18]. Toshimasa Hayashi successfully identified two clinical isolates of Streptococcus suis (S. suis) in blood samples obtained from two male farmers in Japan using next generation sequencing (NGS), and further confirmed that the two isolates belonged to serotype 2 ST28 [13]. It was also reported that a male patient working as a butcher who injured his finger while handling pork developed sepsis. However, no pathogenic bacteria were found in common blood culture examination. S. suis was confirmed by macrogene second-generation sequencing using blood samples [19]. A critical review of literature revealed that few studies have reported the application of mNGS-based detection to determine the presence of the pathogens in patient blood samples.

To the best of our knowledge, the use of cerebrospinal fluid metagenomic next generation sequencing for the diagnosis of Streptococcus suis meningoencephalitis has been rarely reported. A case of Streptococcus suis meningoencephalitis was diagnosed by next generation sequencing of cerebrospinal fluid metagenome in our department. Initially, mNGS was used to diagnose central nervous system infections, such as chronic infections, and it has since been used to successfully diagnose rare encephalitis, new encephalitis, and atypical infections [8, 16, 20]. For cases of encephalitis with unknown cause, or atypical symptoms, mNGS may be more effective than conventional methods like CSF smear and culture [8, 14, 15].

Currently, penicillin, ampicillin and 3rd or 4th generation cephalosporin are the most effective agents against S. suis [21]. The patient in this case report had high fever and consciousness disturbance at the time of admission. Cytological and biochemical CSF analyses lead us to conclude that this was a case of severe bacterial meningeal encephalitis. At admission, the patient was treated with meropenem, a strong broad-spectrum antibiotic that penetrates the blood-brain barrier. Five days after admission, second-generation sequencing of the patient’s CSF sample confirmed S. suis meningoencephalitis. About 1 week after admission, the patient’s symptoms and CSF laboratory test results had improved significantly. Thus, we changed treatment to ceftazidime until routine and biochemical CSF test results were normal.

This study indicates that mNGS analysis of CSF is an “unbiased” pathogen detection method for accurate clinical diagnosis of patients suspected of nervous system infection, even in cases of rare pathogen infection [8, 9]. Because mNGS can simultaneously examine nucleic acid sequences of a variety of pathogenic microorganisms, including common and rare bacteria, it provides high detection rates, facilitating early initiation of treatment. This improves patient prognosis and minimizes use of non-targeted antibiotics.

## Data Availability

The data that support the findings of the current study are available from the corresponding author upon reasonable request.

## Abbreviations

CSF: Cerebrospinal fluid
NGS: Next-generation sequencing
mNGS: Metagenomic next-generation sequencing
S.suis: Streptococcus suis
